# A Catalogue of Plants in the Vicinity of Columbus, Ohio

**Published:** 1840

**Authors:** 


Art. XVIII.—*A Catalogue of Plants, Native and Naturalized, in the vicinity of Columbus, Ohio.* By Wm. S. Sullivant, 1840, p. 63.

Why do so few of our young physicians, a vast majority of whom practise medicine in the country, devote themselves to the study of Botany? Can not nature, with her brilliant hues and balmy odors, captivate them? Do they not appreciate the fact, that new discoveries might be made, and their names handed down to posterity? Do they recognize no relations between Botany and Materia Medica? Between vegetable and animal Physiology? We have always marvelled, that, living in the depths of a wilderness but half subdued, so small a number of students of medicine, and of our young men of wealth and leisure generally, should turn to the cultivation of this delightful science. Thirty years ago, there was an excuse for this neglect, in the difficulty of obtaining suitable books—either elementary or practical; but that difficulty no longer exists; and for a few dollars, an adequate library may be obtained. The author of the pamphlet before us, is a gentleman of leisure, but not of laziness; and although not under the necessity of being active in business, is active—even laborious, in collecting and studying the plants of his neighbourhood. Would that more of our sons possessed his taste.

Mr. Sullivant has arranged his plants on the natural method of Lindley, which may be studied in the "Introduction to the Natural System of Botany," of that distinguished Botanist, edited by Torrey and republished in New York. Of the two hundred and seventy-two natural orders in that system, the catalogue of Mr. S. embraces one hundred and twenty-seven, of which but two are exclusively exotic. This, considering the narrow limits of his locality, seems very remarkable ; and depends, perhaps, first on its latitude, about 40° N., combining the climatorial influences of the temperate zone : and second, on its geological character. This is essentially diluvial, though there are tracts in which a compact secondary lime-stone shows itself. The diluvium has evidently been brought from the distant north, as much of it is primitive, and it may be traced towards the lakes ; which, it is clear, once discharged great quantities of water over the summit levels of Ohio, Indiana, and Illinois.

Among the native genera of this catalogue, we notice more than forty, which embrace species, known to be medicinal or poisonous—if the distinction may be allowed. But how little do we know of the component parts, and action on the living body, of our native plants. As yet, a most superficial examination is all that the majority of them has received ; and there the physicians of the United States seem to have stopped.

A few monographs, botanical, pharmaceutic and chemical, have, it is true, been put forth, but the work, has in fact, only been commenced. This is the inevitable effect, of the slight chemical and botanical training to which our students of medicine are subjected.

We trust that Mr. S. will continue his labours, gradually extending his field of observation, and that many other floras will at no distant time, be the consequence of his example. It is only by their aid, that a general and complete catalogue of our plants can be made out. Such a work is much wanted, and we are happy to know that our confrere, Professor Short, is preparing such a work for the press. His personal researches for twenty-five years, have eminently prepared him for giving to the world a flora of the central parts of the United States.

We ought to state that Mr. S. is desirous of exchanging specimens, with other botanists: and that his brother Mr. John Sullivant, who is devoted to the study of the mineralogy and organic remains, of the west, will be pleased to make similar exchanges with other naturalists. D.

